# Somatic mutations in endometrial epithelium: biological insights and emerging *in vitro* models

**DOI:** 10.1093/molehr/gaag027

**Published:** 2026-05-09

**Authors:** Sugarniya Subramaniam, Caroline E Gargett, Harriet C Fitzgerald, Grant W Montgomery, Brett McKinnon

**Affiliations:** Institute for Molecular Bioscience, The University of Queensland, St Lucia, Brisbane, QLD, Australia; The Ritchie Centre, Hudson Institute of Medical Research, Clayton, VIC, Australia; Department of Obstetrics and Gynaecology, Monash University, Clayton, VIC, Australia; The Ritchie Centre, Hudson Institute of Medical Research, Clayton, VIC, Australia; Department of Obstetrics and Gynaecology, Monash University, Clayton, VIC, Australia; Institute for Molecular Bioscience, The University of Queensland, St Lucia, Brisbane, QLD, Australia; Institute for Molecular Bioscience, The University of Queensland, St Lucia, Brisbane, QLD, Australia

**Keywords:** endometrium, stem cell, endometriosis, mutation, *in vitro* models, epithelial cells, lesion, organoid

## Abstract

The human endometrium is a complex, dynamic, and poorly understood tissue involving monthly cyclical regeneration likely from adult stem/progenitor cells. This regeneration is associated with reproductive pathologies, such as endometriosis and adenomyosis. Endometrial epithelial cells have rates of mutations that are higher than expected for non-malignant tissue. Many of these mutations occur in known cancer-driver genes and can be inherited by endometriosis or adenomyosis lesions. The impact of these mutations on endometrial function and their contribution to endometrial pathologies is unclear. This gap in knowledge is partly due to the lack of suitable *in vitro* models for studying their effects on endometrial epithelial function. In this manuscript, we present a narrative review of the mutation landscape of the endometrium, their potential means of acquisition, and methods to study their consequences. Cataloguing and studying the role of somatic mutations will help us to understand their contribution to endometrial disease. Identifying their molecular and functional consequences will improve diagnostics, facilitate targeted treatment, and provide a step towards the personalization of treatment for endometrial pathologies.

## Graphical Abstract

**Graphical Abstract:**
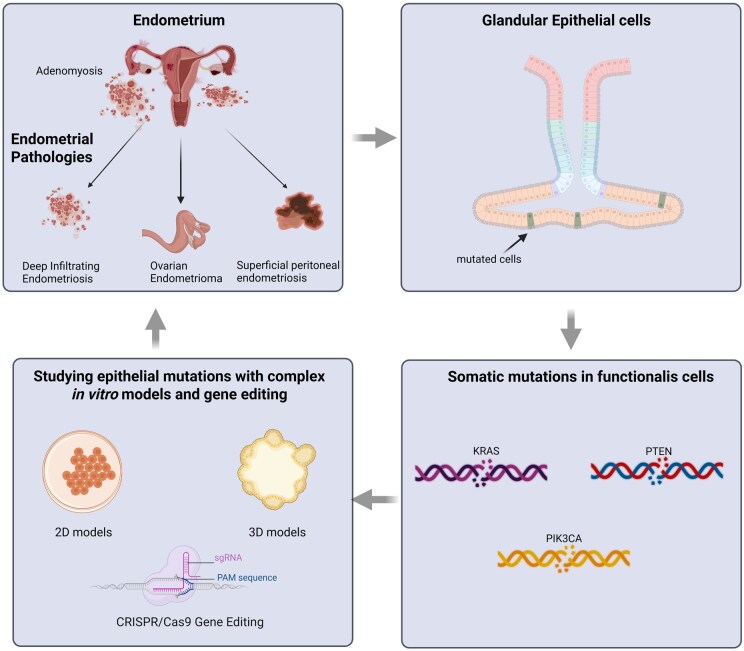
**Somatic mutations of endometrial tissues are studied using *in vitro* models for their potential role(s) in personalized therapy**. Created in BioRender. Subramaniam, S. (2026) https://BioRender.com/bsj0lby.

## Endometrial epithelial cells, their development, roles and functions

**Figure 1.**
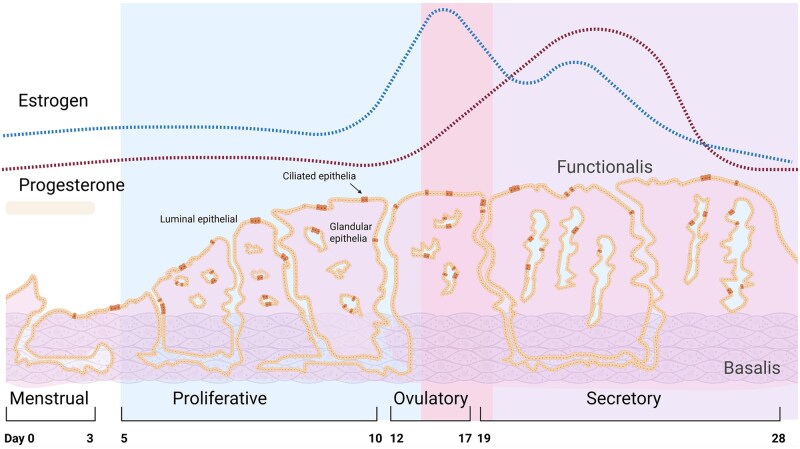
**Shedding, growth and differentiation of endometrial epithelial cells**. The endometrium consists of the basalis and functionalis layer, with the basalis remaining throughout the menstrual cycle, while the functionalis is regenerated from the epithelial stem/progenitor cells residing in the horizontal branching glands of the basalis. Basalis SSEA-1+ epithelial cells from gland stumps migrate over the denuded endometrium to re-epithelialise it or repair it, thereby generating the new luminal epithelium. During the proliferative stage, estrogen dominance drives the rapid proliferation of the glandular and luminal epithelial cells (transit amplifying cells) of the vertical glands as they extend vertically into the functionalis, to generate a mucosa up to 10 mm in thickness. Multiple vertical glands can emanate from the same horizontal glandular segments, indicating that mature independent glands share common cellular origins. In the secretory phase under progesterone dominance, the epithelial cells of the glands differentiate into secretory cells producing a histiotroph for nourishing an implanting blastocyst until placentation is fully established. Differentiated ciliated epithelial cells are distributed in both the glandular and luminal epithelium. Created in BioRender. Subramaniam, S. (2026) https://BioRender.com/6ps9y39.

The human endometrium is a dynamic and regenerative tissue. It renews monthly through cyclical regeneration from adult stem/progenitor cells and is associated with reproductive pathologies, such as endometriosis and adenomyosis. The human endometrium is the lining of the uterus and originally derived from embryonic mesoderm (Ye *et al.*, 2011). Morphologically, it comprises two layers: the basalis and functionalis (Fig. 1). The thin deep germinal basalis layer remains from cycle to cycle while the upper functionalis layer is highly regenerative (Jabbour *et al.*, 2006). Towards the end of the menstrual cycle, apoptosis can be observed, and shedding of the functionalis occurs. The process of endometrial shedding starts superficially and gradually progresses to the deeper layers, lasting for 3–5 days, after which regeneration commences from the basal layer (Maybin and Critchley, 2015).

During regeneration, endometrial epithelial cells acquire mutations at rates higher than those observed for stromal cells and higher than what would normally be expected for epithelium (Suda *et al.*, 2019). These mutations are largely confined to epithelial cells. In micro-dissected endometriotic lesions, somatic mutations were observed to be significantly enriched in the epithelium and not in the stromal component (Noe *et al.*, 2018). Whole‑exome and targeted sequencing studies of deep infiltrating endometriosis (DIE) also showed that known cancer driver mutations were limited to the epithelial cells, with no detectable mutations in the stromal cells of the same lesions (Anglesio *et al.*, 2017). However, evidence from a recent study suggests that, while uncommon, somatic mutations can also be detected in the stromal compartment, indicating that stromal contribution may not be entirely absent (Olafsson *et al.*, 2026).

Together, these findings support epithelial cells as the primary carriers of somatic alterations in endometriosis, whereas stromal cells do not show the same frequency of mutations. Extensive proliferation of epithelial cells that harbour mutations within individual glands may result in regions of the endometrium with an altered genetic profile, potentially impacting the function of otherwise normal glands or contributing to the development of pathology. The role these mutations play in endometrial pathologies is not yet clear, but deserves further attention. Historically, we have had a limited ability to study endometrial epithelial function and the implications of genetic alterations, but this is changing with development of new tools and approaches.

### Epithelial progenitor cells

In human endometrium, the regenerative capacity is facilitated by stem/progenitor cells located in the basalis endometrium (Gargett, 2004, 2007). These epithelial progenitor cells, identified by the N-cadherin marker in the horizontal branching glands of the deep basalis, are quiescent and rarely proliferate, despite expressing estrogen receptor alpha (ESR1) (Valentijn *et al.*, 2013; Nguyen *et al.*, 2017) (Fig. 1). In the early proliferative stage, glands emanate from horizontal basalis gland segments (Yamaguchi *et al.*, 2021), generating the vertical glands which contain the dividing epithelium of the functionalis glands (Fig. 1). At this stage, the functionalis epithelial glands are straight, narrow, and tubular, and lined with low columnar epithelium. With each round of cell division, the epithelial cells become more differentiated and gradually lose their proliferative capacity, as the vertical glands grow and give rise to the luminal epithelium (Gargett, 2007). This differentiation occurs under the influence of progesterone during the secretory phase (Gargett *et al.*, 2008). If no embryo is present, or if the embryo does not implant or is developmentally compromised (Norwitz *et al.*, 2001), the progesterone levels fall, and a new cycle commences with the shedding of the functionalis layer during menstruation.

New insights into the structure of the basalis glands, from lineage tracing of mitochondrial DNA mutations (Tempest *et al.*, 2020) and by tissue clearing and 3D imaging, have revealed horizontal, rhizome-like glandular structures that overlap and extend across multiple regions to provide deep connections between otherwise independent glands (Yamaguchi *et al.*, 2022). Multiple vertical glands emanate from the same horizontal glandular segment, indicating that mature independent glands share common cellular origins in this basalis structure (Fig. 1).

### Mature epithelial cells

As the proliferation continues, both luminal and glandular epithelium form the new functionalis (Fig. 1). These epithelial cells develop from the basalis epithelial progenitors through a coordinated exposure to estrogen and progesterone (Maybin and Critchley, 2015). Estrogen secreted by the developing ovarian follicle binds to ESR-1 from the endometrial stromal cells, inducing the proliferation of N-cadherin-negative glandular epithelial cells in the nascent vertical glands (Cunha *et al.*, 2017; Nguyen *et al.*, 2017). The precise timing of when the endometrial epithelial cells become responsive to estrogen remains unknown.

The luminal epithelial lining the inner surface of the uterus is the site of initial attachment and implantation of the embryo (Fukui *et al.*, 2019). Tall columnar luminal epithelial cells, originally derived from the basalis gland epithelium, cover the surface of the uterine lumen, serving as a barrier which separates the uterine contents from the underlying tissue. After ovulation, proliferation slows, and in response to progesterone, the luminal epithelial cells change their morphology to short columnar or cuboidal cells, and the glandular cells differentiate into secretory cells, which secrete histotrophic factors essential for embryo implantation and conceptus growth (Hempstock *et al.*, 2004; Sternberg *et al.*, 2021).

### Ciliated lineages

Epithelial proliferation is accompanied by ciliogenesis of both the glandular and luminal cells (Ludwig and Metzger, 1976) as they differentiate (Fig. 1), displaying distinct transcriptomic profiles (Wang *et al.*, 2020; Garcia-Alonso *et al.*, 2021). Ciliated epithelial cells are characterized by the presence of motile cilia that contribute to fluid flow and cellular locomotion. Variations in the proportion of ciliated cells in the lumen and glandular regions of the endometrium have been reported across time during the menstrual cycle, peaking in both compartments at about 20% of the cells (Masterton *et al.*, 1975) and decreasing during pregnancy and after hormonal treatment (Brosens and Vasquez, 1976; Verhage *et al.*, 1979).

## Somatic mutations in endometrial epithelial cells

The cyclical replication of epithelial cells, as required for the regenerative process, increases the chance of random genetic errors arising in the daughter cells. These could be propagated through subsequent cycles or inherited by non-shedding cells within ectopic endometrial tissues, such as lesions of endometriosis or adenomyosis. Evidence supports an increased incidence of mutations in epithelial cells in both normal endometrium and in benign lesions (Suda *et al.*, 2019). Whether this high mutation rate contributes to these pathologies is an open question; however, the presence in both eutopic and ectopic epithelial cells are increasingly being catalogued (Table 1).

**Table 1.** Somatic mutations observed in endometrial and endometriotic tissue.

Gene	SUP	OMA	Atypical	DIE	Incisional	Adenomyosis	Endometrium	Tissue source	Compartment	Method	Reference	Canonical function	Functional consequences of mutation
*ACRC*		3/45					0/29	Excised lesion and LCM/Hysterectomy and size filtration	Epithelial	Targeted sequencing panel	Suda *et al.* (2018)	DNA–protein cross‑link repair, supporting genomic stability	Limited functional data
*AKT1*							0/25	Hysterectomy and macro dissection	Epithelial	FIND IT targeted sequencing	Lac *et al.* (2019)	Mediates PI3K-dependent signaling, promoting metabolism, proliferation, cell survival, growth and angiogenesis	Promotes cell growth
*AKT1*							1/85	Endometrial biopsy	Whole endometrium	FIND IT targeted sequencing	Lac *et al.* (2019)		
*APC*						1/17		FFPE tissue cores	Adenomyosis foci	Targeted panel sequencing	Chao *et al.* (2023)	Regulates Wnt signaling, cell migration and adhesion, transcriptional activation, and apoptosis.	WNT/β-catenin pathway dysregulation
*ARHGAP35*		1/13					3/11	Excised lesion and LCM/Hysterectomy and size filtration	Epithelial	Whole exome sequencing	Suda *et al.* (2018)	Inactivates RhoA, regulating cell differentiation, adhesion and migration	Rho GTPase deregulation
*ARHGAP35*		2/45					13/29	Excised lesion and LCM/Hysterectomy and size filtration	Epithelial	Targeted sequencing panel	Suda *et al.* (2018)		
*ARHGAP35*							11/28	Hysterectomy/Biopsy/residual transplant/Autopsy and LCM	Epithelial	Whole genome sequencing	Moore *et al.* (2020)		
*ARHGAP35*							2/20	Hysterectomy/laser capture	Epithelial	Whole exome sequencing	Li *et al.* (2021)		
*ARID1A*	0/1		2/2					Excised lesion/LCM	Epithelial	Sanger sequencing on ARID1A codon IHC on BAF250a	Wiegand *et al.* (2010)	Regulates gene transcription, maintains chromatin structure, and supports DNA repair	Alters transcription
*ARID1A*		2/101						Excised lesion	Whole excised lesions	PCR based targeted Sequencing	Zou *et al*. (2018a)		
*ARID1A*				2/24				FFPE Core sampling	Epithelial	Whole exome sequencing/IHC	Anglesio *et al.* (2017)		
*ARID1A*	1/2							Manual Macro dissection	Epithelial	Whole exome Sequencing	Kurose *et al.* (2021)		
*ARID1A*							0/25	Hysterectomy	Epithelial	Loss of IHC expression	Lac *et al.* (2019)		
*ARID1A*							0/85	Endometrial biopsy	Whole endometrium	Loss of IHC expression	Lac *et al.* (2019)		
*ARID1A*				1/36	0/40			Marco dissection/LCM	Epithelial	Loss of expression in IHC	Lac *et al.* (2019)		
*ARID1A*						2/17		FFPE tissue cores	Adenomyosis foci	Targeted panel sequencing	Chao *et al.* (2023)		
*ARID1A*		7/45					0/29	Excised lesion and LCM/Hysterectomy and size filtration	Epithelial	Targeted Sequencing panel	Suda *et al*. (2018)		
*ARID1B*						1/17		FFPE tissue cores	Adenomyosis foci	Targeted panel sequencing	Chao *et al.* (2023)	Transcriptional activation and repression by chromatin remodeling	Alters transcription
*ARID5B*							1/28	Hysterectomy/Biopsy/residual transplant/Autopsy and LCM	Epithelial	Whole genome sequencing	Moore *et al.* (2020)	Modulates gene expression by binding AT-rich DNA elements	Alters transcription
*ATM*							1/28	Hysterectomy/Biopsy/residual transplant/Autopsy and LCM	Epithelial	Whole genome sequencing	Moore *et al.* (2020)	Detects DNA double-strand breaks, activates DNA repair and cell cycle checkpoints	Genomic instability
*ATRX*				1/13			1/13	Excised lesions and LCM/Hysterectomy	Epithelial	Targeted sequencing of 1296 genes	Koppolu *et al.* (2021)	Transcriptional regulation and chromatin remodeling	Genomic instability
*BRCA1*						1/20		Hysterectomy/laser capture	Epithelial	Whole exome sequencing	Li *et al.* (2021)	Maintains genomic stability	Genomic instability
*BRAF*		0/101						Excised lesion	Whole excised lesions	PCR-based targeted Sequencing	Zou *et al*. (2018a)	Regulates MAP kinase/ERK signaling pathway, cell division, differentiation, and secretion	Alters cell survival and proliferation
*BRAF*							1/28	Hysterectomy/Biopsy/residual transplant/Autopsy and LCM	Epithelial	Whole genome sequencing	Moore *et al.* (2020)		
*CARD10*		4/101						Excised lesions	Whole lesion	PCR-based targeted sequencing of the whole gene	Zou *et al.* (2018b)	Member of membrane-associated guanylate kinase family and activates NF-kappa-B	Loss leads to cell proliferation and increase inflammation
*CARD11*		2/101						Excised lesions	Whole lesion	PCR-based targeted sequencing of the whole gene	Zou *et al.* (2018b)	Role in adaptive immune response	Alters immune signalling pathways
*CDH4*							6/28	Hysterectomy/Biopsy and LCM	Epithelial	Whole genome sequencing	Moore *et al.*, (2020)	Plays a key role in cell adhesion	Drives stemness
*CDH4*	1/12							Hysterectomy/laser capture	Epithelial	Whole exome sequencing	Li *et al.* (2021)		
*CDKN1B*							1/28	Hysterectomy/Biopsy/residual transplant/Autopsy and LCM	Epithelial	Whole genome sequencing	Moore *et al.* (2020)	Encodes a cyclin-dependent kinase inhibitor.	Leads to cell proliferation
*CREBBP*							2/28	Hysterectomy/Biopsy/residual transplant/Autopsy and LCM	Epithelial	Whole genome sequencing	Moore *et al.* (2020)	Transcription factors to regulate gene expression,	Alters cell growth
*CTCF*		2/92						Excised lesion	Whole tissue	PCR-based sequencing of whole codon	Guo *et al*. (2018)	Transcriptional activator of a histone deacetylase containing complex	Alters transcription
*CTNNB1*				1/36	0/40			Marco dissection/LCM	Epithelial	FIND IT targeted sequencing	Lac *et al.* (2019)	A key transcriptional effector of Wnt signaling and a structural component of adherens junctions	Drives proliferation
*CTNNB1*	1/12							Hysterectomy/laser capture	Epithelial	Whole exome sequencing	Li *et al.* (2021)		
*CTNNB1*		3/45					1/29	Excised lesion and LCM/Hysterectomy and size filtration	Epithelial	Targeted sequencing panel	Suda *et al.* (2018)		
*DICER*						1/17		FFPE tissue cores	Adenomyosis foci	Targeted panel sequencing	Chao *et al.* (2023)	A ribonuclease regulating gene expression	Deregulation of gene expression
*DNAH7*				2/13				Excised lesions and LCM/Hysterectomy	Epithelial	Targeted sequencing of 1296 genes	Koppolu *et al.* (2021)	A role in respiratory cilia	Limited functional data
*DNMT3A*						1/17		FFPE tissue cores	Adenomyosis foci	Targeted panel sequencing	Chao *et al.* (2023)	Encodes a DNA methyltransferase	Alters gene expression
*EGFR*						1/17		FFPE tissue cores	Adenomyosis foci	Targeted panel sequencing	Chao *et al.* (2023)	A receptor tyrosine kinase that binds growth factors	Leads to cell proliferation
*ERBB2*				1/36	0/40			Marco dissection/LCM	Epithelial	FINDIT targeted sequencing	Lac *et al.* (2019)	Activates MAPK and PI3K-AKT signaling, regulating cell proliferation and survival	Leads to cell proliferation
*ERBB2*							1/25	Hysterectomy and macro dissection	Epithelial	FIND IT targeted sequencing	Lac *et al.* (2019)		
*ERBB2*							0/85	Endometrial biopsy	Whole endometrium	FIND IT targeted sequencing	Lac *et al.* (2019)		
*ERBB2*							5/28	Hysterectomy/Biopsy/residual transplant/Autopsy and LCM	Epithelial	Whole genome sequencing	Moore *et al.* (2020)		
*ERBB3*							3/28	Hysterectomy/Biopsy/residual transplant/Autopsy and LCM	Epithelial	Whole genome sequencing	Moore *et al.* (2020)	Activates PI3K-AKT and MAPK pathways, regulating cell proliferation or differentiation	Leads to cell proliferation
*ERBB3*						1/20		Hysterectomy/laser capture	Epithelial	Whole exome sequencing	Li *et al.* (2021)		
*ERK1*		0/101						Excised lesion	Whole excised lesions	PCR-based targeted sequencing	Zou *et al*. (2018a)	Essential component of the MAP kinase signal transduction pathway.	Leads to cell proliferation
*ERK2*		0/101						Excised lesion	Whole excised lesions	PCR-based targeted sequencing	Zou *et al*. (2018a)	Essential component of the MAP kinase signal transduction pathway.	Leads to cell proliferation
*EYS*							1/20	Hysterectomy/laser capture	Epithelial	Whole exome sequencing	Li *et al.* (2021)	Maintains the integrity of photoreceptor cells	Limited functional data
*FAT1*							1/28	Hysterectomy/Biopsy/residual transplant/Autopsy and LCM	Epithelial	Whole genome sequencing	Moore *et al.* (2020)	Mediates cell–cell adhesion, regulates planar cell polarity, Modulates Wnt/Hippo signaling, and cytoskeletal dynamics.	Leads to cell proliferation
*FAT1*		4/45					2/29	Excised lesion and LCM/Hysterectomy and size filtration	Epithelial	Targeted sequencing panel	Suda *et al.* (2018)		
*FBN2*		2/13					1/11	Excised lesion and LCM/Hysterectomy and size filtration	Epithelial	Whole exome sequencing	Suda *et al.* (2018)	Essential role in cellular polarization, directed cell migration, and cell communication	Limited functional data
*FBXW7*		1/13					4/11	Excised lesion and LCM/Hysterectomy and size filtration	Epithelial	Whole exome sequencing	Suda *et al.* (2018)	Ubiquitin-mediated degradation and controlling cell cycle	Alters cell cycle
*FBXW7*		1/45					6/29	Excised lesion and LCM/Hysterectomy and size filtration	Epithelial	Targeted sequencing panel	Suda *et al.* (2018)		
*FBXW7*							8/28	Hysterectomy/Biopsy/residual transplant/Autopsy and LCM	Epithelial	Whole genome sequencing	Moore *et al.* (2020)		
*FBXW7*						1/17		FFPE tissue cores	FFPE tissue cores	Targeted panel sequencing	Chao *et al.* (2023)		
*FBXW7*	1/12					1/20		Hysterectomy/laser capture	Epithelial	Whole exome sequencing	Li *et al.* (2021)		
*FGFR1*						1/17		FFPE tissue cores	FFPE tissue cores	Targeted panel sequencing	Chao *et al.* (2023)	A role in regulation of embryonic development, cell proliferation, differentiation and migration	Promotes proliferation
*FGFR2*							2/25	Hysterectomy	Epithelial	Loss of IHC expression	Lac *et al.* (2019)	A role in regulation of cell proliferation, migration, differentiation and apoptosis, and embryonic development	Promotes proliferation
*FGFR2*							4/85	Endometrial biopsy	Whole endometrium	Loss of IHC expression	Lac *et al.* (2019)		
*FGFR2*							3/28	Hysterectomy/Biopsy/residual transplant/Autopsy and LCM	Epithelial	Whole genome sequencing	Moore *et al.* (2020)		
*FGFR2*		2/45					5/29	Excised lesion and LCM/Hysterectomy and size filtration	Epithelial	Targeted sequencing panel	Suda *et al*. (2018)		
*FOXA2*							7/28	Hysterectomy/Biopsy/residual transplant/Autopsy and LCM	Epithelial	Whole genome sequencing	Moore *et al.* (2020)	A transcription factor involved in embryonic development.	Regulates lesion formation
*FRG1*		2/13					1/11	Hysterectomy/Biopsy/residual transplant/Autopsy and LCM	Epithelial	Whole GENOME sequencing	Suda *et al*. (2018)	RNA processing	Limited functional data
*GNAS*						1/17		FFPE tissue cores	FFPE tissue cores	Targeted panel sequencing	Chao *et al.* (2023)	Regulation of pre-mRNA splicing and the assembly of rRNA	Limited functional data
*HEATR1*		3/13					0/11	Excised lesion and LCM/Hysterectomy and size filtration	Epithelial	Whole exome sequencing	Suda *et al*. (2018)	Ribosome biogenesis and the regulation of RNA metabolic processes.	Limited functional data
*HEATR1*		3/45					5/29	Excised lesion and LCM/Hysterectomy and size filtration	Epithelial	Targeted sequencing panel	Suda *et al*. (2018)		
*HRAS*		0/101						Excised lesion	Whole excised lesions	PCR-based targeted sequencing	Zou *et al*. (2018a)	Activation of Ras protein signal transduction	Limited functional data
*HRAS*							1/28	Hysterectomy/Biopsy/residual transplant/Autopsy and LCM	Epithelial	Whole genome sequencing	Moore *et al.* (2020)		
*KIAA1109*		2/13					1/11	Excised lesion and LCM/Hysterectomy and size filtration	Epithelial	Whole exome sequencing	Suda *et al.* (2018)	Vesicular trafficking and intracellular organization and cellular homeostasis	Limited functional data
*KMT2C*							2/28	Hysterectomy/Biopsy/residual transplant/Autopsy and LCM	Epithelial	Whole genome sequencing	Moore *et al.* (2020)	Transcription regulation	Transcription dysregulation
*KMT2C*		3/45					2/29	Excised lesion and LCM/Hysterectomy and size filtration	Epithelial	Targeted sequencing panel	Suda *et al*. (2018)		
*KMT2D*							1/28	Hysterectomy/Biopsy/residual transplant/Autopsy and LCM	Epithelial	Whole genome sequencing	Moore *et al.* (2020)	A histone methyltransferase	Transcription dysregulation
*KRAS*		8/13					4/11	Excised lesion and LCM/Hysterectomy and size filtration	Epithelial	Whole exome sequencing	Suda *et al.* (2018)	Regulation of cell proliferation	P(romotes )proliferation
*KRAS*		17/45					7/29	Excised lesion and LCM/Hysterectomy and size filtration	Epithelial	Targeted sequencing panel	Suda *et al.* (2018)		
*KRAS*		1/101						Excised lesion	Whole excised lesions	PCR based targeted sequencing	Zou *et al*. (2018a)		
*KRAS*		6/20						FFPE section of whole lesion	Whole lesion	Sanger sequencing	Yachida *et al.* (2021)		
*KRAS*		7/12						LCM	Epithelial	Targeted sequencing/In situ hybridization	Yachida *et al.* (2021)		
*KRAS*	0/14	0/10		0/13				Microdissection of epithelial glands	Excised glands	PCR-based sequencing of codon 12 and 13	Kim *et al.* (2018)		
*KRAS*				7/36	2/40			Marco dissection/LCM	Epithelial	FIND IT targeted sequencing/Digital droplet PCR	Lac *et al.* (2019)		
*KRAS*							8/25	Hysterectomy	Epithelial	Loss of IHC expression	Lac *et al.* (2019)		
*KRAS*							23/85	Endometrial biopsy	Whole endometrium	Loss of IHC expression	Lac *et al.* (2019)		
*KRAS*							4/28	Hysterectomy/Biopsy/residual transplant/Autopsy and LCM	Epithelial	Whole genome sequencing	Moore *et al.* (2020)		
*KRAS*				1/24				No separation	Whole lesion	Whole exome sequencing	Anglesio *et al.* (2017)		
*KRAS*				5/14				Macro dissection	Whole lesion	digital droplet PCR	Anglesio *et al.* (2017)		
*KRAS*						26/70		Multi region sampling	Whole tissue	Whole genome sequencing/targeted sequencing	Inoue *et al.* (2019)		
*KRAS*						4/17		FFPE tissue cores	Adenomyosis foci	Targeted panel sequencing	Chao *et al.* (2023)		
*NF1*							2/28	Hysterectomy/Biopsy/residual transplant/Autopsy and LCM	Epithelial	Whole genome sequencing	Moore *et al.* (2020)	Negatively regulates Ras signaling pathways	Promotes proliferation
*NOTCH2*							1/28	Hysterectomy/Biopsy/residual transplant/Autopsy and LCM	Epithelial	Whole genome sequencing	Moore *et al.* (2020)	Regulates transcriptional programs controlling cell fate decisions	Promotes proliferation
*NRAS*		0/101						Excised lesion	Whole excised lesions	PCR-based targeted Sequencing	Zou *et al*. (2018a)	Regulates intracellular signaling pathways	Promotes proliferation
*NRAS*							0/25	Hysterectomy	Epithelial	Loss of IHC expression	Lac *et al.* (2019)		
*NRAS*							2/85	Endometrial biopsy	Whole endometrium	Loss of IHC expression	Lac *et al.* (2019)		
*PIK3CA*		3/13					8/11	Excised lesion and LCM/Hysterectomy and size filtration	Epithelial	Whole exome sequencing	Suda *et al.* (2018)	Activates signaling pathways controlling cell growth, proliferation, survival.	Enhances growth
*PIK3CA*		13/45					12/29	Excised lesion and LCM/Hysterectomy and size filtration	Epithelial	Targeted sequencing panel	Suda *et al.* (2018)		
*PIK3CA*		0/101						Excised lesion	Whole excised lesions	PCR-based targeted Sequencing	Zou et al. (2018a)		
*PIK3CA*				1/24				No separation	Whole lesion	Whole exome sequencing	Anglesio *et al.* (2017)		
*PIK3CA*				0/36	1/40			Marco dissection/LCM	Epithelial	FINDIT targeted sequencing	Lac *et al.* (2019)		
*PIK3CA*							4/25	Hysterectomy	Epithelial	Loss of IHC expression	Lac *et al.* (2019)		
*PIK3CA*							10/85	Endometrial biopsy	Whole endometrium	Loss of IHC expression	Lac *et al.* (2019)		
*PIK3CA*							15/28	Hysterectomy/Biopsy/residual transplant/Autopsy and LCM	Epithelial	Whole genome sequencing	Moore *et al.* (2020)		
*PIK3CA*						2/70		Multi region sampling	Whole tissue	Whole genome sequencing/targeted sequencing	Inoue *et al.* (2019)		
*PIK3R1*		1/13					2/11	Excised lesion and LCM/Hysterectomy and size filtration	Epithelial	Whole exome sequencing	Suda *et al.* (2018)	Regulates cell proliferation, survival, and growth signaling	Enhances cell proliferation
*PIK3R1*		2/45					13/29	Excised lesion and LCM/Hysterectomy and size filtration	Epithelial	Targeted sequencing panel	Suda *et al.* (2018)		
*PIK3R1*							7/28	Hysterectomy/Biopsy/residual transplant/Autopsy and LCM	Epithelial	Whole GENOME sequencing	Moore *et al.* (2020)		
*PIK3R1*						1/20		Hysterectomy/laser capture	Epithelial	Whole exome sequencing	Li *et al.* (2021)		
*PLCG1*							1/28	Hysterectomy/Biopsy/residual transplant/Autopsy and LCM	Epithelial	Whole genome sequencing	Moore *et al.* (2020)	Regulates intracellular signaling cascades controlling actin cytoskeleton reorganization, and cell migration.	Limited functional data
*PLXNB2*		1/13					3/11	Excised lesion and LCM/Hysterectomy and size filtration	Epithelial	Whole exome sequencing	Suda *et al.* (2018)	Mediates semaphorin	Alters epithelial integrity
*PLXNB2*		1/45					5/29	Excised lesion and LCM/Hysterectomy and size filtration	Epithelial	Targeted sequencing panel	Suda *et al.* (2018)		
*PPP2R1A*		0/13					3/11	Excised lesion and LCM/Hysterectomy and size filtration	Epithelial	Whole exome sequencing	Suda *et al.* (2018)	Forms the PP2A scaffold complex to regulate protein dephosphorylation	Enhances growth
*PPP2R1A*		1/101						Excised lesion	Whole excised lesions	PCR-based targeted sequencing	Zou *et al*. (2018a)		
*PPP2R1A*				1/24				No separation	Whole lesion	Whole exome sequencing	Anglesio *et al.* (2017)		
*PPP2R1A*							5/28	Hysterectomy/Biopsy/residual transplant/Autopsy and LCM	Epithelial	Whole genome sequencing	Moore *et al.* (2020)		
*PPP2R1A*						1/70		Multi region sampling	Whole tissue	Whole genome sequencing/targeted sequencing	Inoue *et al.* (2019)		
*PRDM1*							1/28	Hysterectomy/Biopsy/residual transplant/Autopsy and LCM	Epithelial	Whole genome sequencing	Moore *et al.* (2020)	A transcriptional repressor controlling immune cell differentiation	Limited functional data
*PTEN*							2/28	Hysterectomy/Biopsy/residual transplant/Autopsy and LCM	Epithelial	Whole genome sequencing	Moore *et al.* (2020)	A phosphatase that suppresses PI3K/AKT signaling to regulate cell cycle progression and cell survival	Enhances cell growth and reduces apoptosis
*PTEN*				4/36	7/40			Marco dissection/LCM	Epithelial	Loss of expression in IHC	Lac *et al.* (2019)		
*PTEN*	1/2							Manual Macro dissection	Epithelial	Whole exome sequencing	Kurose *et al.* (2021)		
*PTEN*							10/25	Hysterectomy	Epithelial	Loss of IHC expression	Lac *et al.* (2019)		
*PTEN*							20/85	Endometrial biopsy	Whole endometrium	Loss of IHC expression	Lac *et al.* (2019)		
*PTEN*		0/101						Excised lesion	Whole excised lesions	PCR-based targeted Sequencing	Zou *et al*. (2018a)		
*PTEN*		2/45					3/29	Excised lesion and LCM/Hysterectomy and size filtration	Epithelial	Targeted sequencing panel	Suda *et al.* (2018)		
*PTPN13*		1/13					2/11	Excised lesion and LCM/Hysterectomy and size filtration	Epithelial	Whole exome sequencing	Suda *et al.* (2018)	A tyrosine phosphatase that modulates signaling pathways to regulate mitotic cycle	Limited functional data
*RRAS*							2/28	Hysterectomy/Biopsy/residual transplant/Autopsy and LCM	Epithelial	Whole genome sequencing	Moore *et al.* (2020)	Regulates angiogenesis, vascular homeostasis and axon guidance	Limited functional data
*RYR1*				1/13			1/13	Excised lesions and LCM/Hysterectomy	Epithelial	Targeted sequencing of 1296 genes	Koppolu *et al.* (2021)	Regulates the release of calcium to control skeletal muscle contraction	Limited functional data
*SMAD2*							1/28	Hysterectomy/Biopsy/residual transplant/Autopsy and LCM	Epithelial	Whole genome sequencing	Moore *et al.* (2020)	Regulates transcription programs controlling cell proliferation, apoptosis, and differentiation	Alters epithelial differentiation
*SPOP*							2/28	Hysterectomy/Biopsy/residual transplant/Autopsy and LCM	Epithelial	Whole GENOME sequencing	Moore *et al.* (2020)	Mediates ubiquitination of target proteins via the Cullin3 E3 ligase	Loss leads to the accumulation of oncogenic proteins
*SPOP*						1/17		FFPE tissue cores	Adenomyosis foci	Targeted panel sequencing	Chao *et al.* (2023)		
*STAG2*							1/28	Hysterectomy/Biopsy/residual transplant/Autopsy and LCM	Epithelial	Whole Genome sequencing	Moore *et al.* (2020)	A cohesion subunit controlling chromatid cohesion, chromosome segregation, and genomic stability	Limited functional data
*STAG2*						1/17		FFPE tissue cores	Adenomyosis foci	Targeted panel sequencing	Chao *et al.* (2023)		
*TAF1*		4/45					3/29	Excised lesion and LCM/Hysterectomy and size filtration	Epithelial	Targeted sequencing panel	Suda *et al*. (2018)	Mediates chromatid cohesion, chromosome segregation, and genomic stability	Limited functional data
*TASR31*		3/13					0/11	Excised lesion and LCM/Hysterectomy and size filtration	Epithelial	Whole EXOME sequencing	Suda *et al.* (2018)	Mediates G protein-coupled second messenger pathway	Limited functional data
*TASR1*		3/45					3/29	Excised lesion and LCM/Hysterectomy and size filtration	Epithelial	Targeted sequencing panel	Suda *et al*. (2018)	Mediates umami taste perception	Limited functional data
*TERT*						1/17		FFPE tissue cores	Adenomyosis foci	Targeted panel sequencing	Chao *et al.* (2023)	A role in cellular senescence and chromosomal repair	Alters telomerase activity
*TP53*	1/2							Manual Macro dissection	Epithelial	Whole exome sequencing	Kurose *et al.* (2021)	Regulates cell cycle, apoptosis, DNA repair and changes in metabolism	Apoptosis resistance
*TP53*							3/28	Hysterectomy/Biopsy/residual transplant/Autopsy and LCM	Epithelial	Whole genome sequencing	Moore *et al.* (2020)		
*TRERF1*		2/92						Excised tissue	Whole lesion	PCR-based sequencing	Cao *et al.* (2018)	Modulates gene expression programs	Limited functional data
*ZFHX3*							3/28	Hysterectomy/Biopsy	Epithelial	Whole genome sequencing	Moore *et al.* (2020)	A transcription factor that modulates myogenic and neuronal differentiation	Limited functional data

• For whole genome and whole exome sequencing studies, genes are only included if specifically addressed by the original article.

• Numbers represent the patients with mutations/number of patients examined.

• If several different techniques were used results of both were included.

SUP, superficial endometriosis; OMA, endometrioma; DIE, deeply infiltrating endometriosis.

### Endometrium

The low frequency of mutations in normal tissue makes their investigation technically challenging. Several strategies to enrich the cells that harbour mutations have been trialled, including sequencing of *in vitro* clones (Blokzijl *et al.*, 2016), the collection of small biopsies containing distinct structural elements (Martincorena *et al.*, 2015, 2018), and the sequencing of single cells (Lodato *et al.*, 2018). Microdissection and targeted gene-sequencing confirmed that endometrial mutations are largely confined to the epithelial compartment (Suda *et al.*, 2019).

Further attempts to enrich the mutation-containing cells by the examination of individual glands revealed a heterogeneous endometrial landscape. Individual glands within the same endometrium display variations in their mutational profile. Whole-exome and targeted sequencing of 11 endometrial glands identified mutations in multiple cancer-associated genes, including phosphatidylinositol-4,5-Bisphosphate 3-Kinase Catalytic Subunit Alpha (*PIK3CA)*, Kirsten Rat Sarcoma Viral Oncogene Homolog *(KRAS)*, Rho GTPase-activating protein 35 *(ARHGAP35)* and Phosphoinositide-3-Kinase Regulatory Subunit 1 *(PIK3RA)* (Suda *et al.*, 2018). Analysis of 257 histologically normal endometrial glands from 28 women found 12 genes that displayed positive selection within glands: *PIK3CA, PIK3R1*, *ARHGAP35*, *F-Box*, and WD Repeat Domain-Containing 7 *(FBXW7)*, Zinc Finger Homeobox 3 *(ZFHX3)*, Forkhead Box A2 *(FOXA2)*, Erb-B2 Receptor Tyrosine Kinase 2 *(ERBB2)*, Chromodomain Helicase DNA-Binding Protein 4 *(CHD4), KRAS*, Speckle-Type POZ Protein *(SPOP)*, Protein Phosphatase 2 Scaffold subunit Alpha *(PPP2R1A)*, and Erb-B2 Receptor Tyrosine Kinase 3 *(ERBB3)* (Moore *et al.*, 2020). Targeted sequencing of 98 women identified mutations in *KRAS* pG12/G13 (59.18%), *PIK3CA* p.H1047 (19.38%), and *PPP2R1A* (15.31%) in normal endometrium and myometrium from women with adenomyosis, with the prevalence being significantly associated with parity (Inoue *et al.*, 2020).

Clonality is a key concept for epithelial mutations to drive endometrial disease. Most genetic alterations are either harmless, producing no influence on phenotype, or so deleterious that cells cannot survive (Lynch *et al.*, 2016). Clonality occurs when there is positive selection of mutant cells that better fit their environment (Watson *et al.*, 2020). In the endometrium, significant clonal expansion likely occurs early in the menstrual cycle with positive selection. Mutations in mature cells are shed during menstruation, whereas mutations acquired early or in the basalis may remain and re-establish clonal glands in the next cycle. This suggests that epithelial mutations acquired in early epithelial cell development may have more severe consequences and lead to clinical manifestations. Identifying recurring molecular alterations in endometrial samples collected over time may help to identify women at risk of benign or malignant conditions. Studies have shown that genomic and epigenetic changes in endometrial tissue may lead to the clinical diagnosis. For example, the presence of aberrant DNA methylation patterns and somatic mutations in benign endometrial biopsies has been shown to correlate with future development of endometrial cancer (Multinu *et al.*, 2020). Another study indicated that molecular analysis of endometrial biofluids may improve the early detection, risk stratification, and monitoring of women with endometrial hyperplasia, who are at risk of developing endometrial cancer (Weng *et al.*, 2022).

Previous studies support the clonal expansion of endometrial glands. Using X chromosome inactivation, a monoclonal composition of endometrial epithelial glands was identified (Tanaka *et al.*, 2003). Analysis of individual glands within the endometrium revealed that 91% of the glands were clonal with a distribution of variant allele frequency (VAF) of between 0.3 and 0.5, suggesting that each gland is descended from a distinct single progenitor stem cell (Moore *et al.*, 2020). A targeted analysis of *KRAS* and *PIK3CA* in ten endometrial glands from three different sections from three different women revealed a varying degree of mutations ranging from 0% to 50% for *PIK3CA* in particular, which indicates clonal expansion (Sato *et al.*, 2023).

### Endometriotic lesions

Endometriosis is the growth of endometrial cells outside the uterine cavity. It is believed to arise from endometrial cells that are refluxed into the peritoneal cavity during menstruation (Sampson, 1927) and this has been demonstrated for human endometrial stem/progenitor cells (Masuda *et al.*, 2021). Stem/progenitor cells, which are shed and transported through retrograde menstruation, are clonogenic and could initiate endometriosis lesions (Cousins *et al.*, 2018). While a molecular-based consensus of endometriosis subtypes is still lacking (International working group of AAGL, ESGE, ESHRE and WES *et al*., 2021), the lesions are currently separated into three groups based on anatomical location and surgical appearance (Chapron *et al.*, 2011): superficial peritoneal lesions (SUP) grow on the lining of the peritoneal cavity; ovarian endometrioma (OMA) are found on the ovaries; and deeply infiltrating endometriosis (DIE) lesions, the most severe form, are characterized by infiltration greater than 5 mm into the underlying tissue.

The first indication that mutations in endometriosis tissue may have consequences for disease progression occurred when targeted mutations of G12D *KRAS* cells in the bursal cavity of BALB/C mice resulted in benign epithelial lesions that closely resembled endometriosis (Dinulescu *et al.*, 2005). Later, a study identified AT Rich Interaction Domain 1A (*ARID1A)* mutations in two atypical endometriosis lesions, contiguous with clear cell ovarian cancer (Wiegand *et al.*, 2010). Subsequent research is beginning to catalogue the incidence and prevalence of mutations in endometriosis across the different anatomical subtypes.

### Deeply infiltrating endometriosis

Parallels have been drawn between DIE, as the most invasive and severe form of the disease, and malignant tissue, leading to the search for somatic driver mutations (Horne and Missmer, 2022). An early study applying exome sequencing on 27 DIE lesions from 24 patients identified mutations in 19 samples (Table 1). Five patients were found to have mutations in cancer driver genes (*ARID1A*, *PIK3CA*, *KRAS*, and *PPP2R1A)* (Anglesio *et al.*, 2017), suggesting they may have a role in establishing non-malignant lesions. A follow-up study of 36 DIE lesions with targeted sequencing of 33 genes identified mutations in *KRAS* (7/36) and Beta-catenin *(CTNNB1) (*1/36) (Lac *et al.*, 2019). Using orthogonal methods, including digital droplet polymerase chain reaction (ddPCR), additional mutations in *ERBB2, PIK3CA*, and *CTNNB1* were observed in the glandular epithelium (Lac *et al.*, 2019) and, finally, with immunohistochemistry, a loss of Phosphatase and Tensin Homolog (PTEN) protein expression in 5 out of 36 patients was also observed (Lac *et al.*, 2019). Altogether this study identified 13 samples of these 36 cases with mutations in cancer driver genes (Lac *et al.*, 2019). In contrast, targeted sequencing of 1,296 genes in the epithelial cells excised from the glandular region of 13 DIE lesions identified only 28 variants, all of which had a low minor allele frequency (MAF) (<10%) (Koppolu *et al.*, 2021). A study of 85 driver genes in a custom-designed panel found mutations in only 5 of the genes (Koppolu *et al.*, 2021). Only one sample had a *KRAS* mutation (p.Gly12Asp) that occurred alongside a mutation in p53 (p.Glu271Lys) (Koppolu *et al.*, 2021). Genes mutated in more than one patient included the passenger genes, Alpha Thalassemia/Mental Retardation Syndrome X-Linked (*ATRX)*, Ryanodine Receptor 1 (*RYR1)*, and Dynein Axonemal Heavy Chain 7 (*DNAH7)* (Koppolu *et al.*, 2021). A focus on *KRAS* codons 12 and 13, the most commonly mutated region of this gene, found no mutations in 13 DIE lesions (Kim *et al.*, 2018). Together, the current literature suggests a complex heterogeneous landscape of mutations in DIE lesions potentially driven by variations in location, age and fibrosis.

### Endometrioma

Endometrioma (OMA) is the growth of endometriotic lesions on the ovary. A spectrum of mutations has also been reported in the epithelial cells within endometrioma, although the genes vary across studies (Table 1). Using a discovery cohort of 13 endometriomas and whole exome sequencing, mutations were observed in 15 genes, including *KRAS (8/13), PIK3CA (3/13)*, Titin *(TTN) (4/13), FBXW7 (1/13), ARHGAP35 (1/13), PPP2R1A (0/13)*, Mucin 6, Oligomeric Mucus/Gel-Forming *(MUC6) (2/13)*, Plexin B2 *(PLXNB2) (1/13)*, CUB and Sushi Multiple Domains 3 *(CSMD3) (1/13)*, Fibrillin 2 *(FBN2) (2/13)*, HEAT repeat containing 1 *(HEATR1) (3/13), KIAA1109 (2/13), PIK3R1 (1/13)*, taste receptor type 2 (*TAS2R31) (3/13)*, Protein Tyrosine Phosphatase, Non-Receptor Type 13 *(PTPN13) (1/13)* and Facioscapulohumeral Muscular Dystrophy Region Gene 1 (*FRG1) (2/13)* (Suda *et al.*, 2018)*. KRAS* was the most frequently mutated gene at hotspot amino acids within codons 12, 13, or 16 (Suda *et al.*, 2018). In contrast, targeted analysis of *KRAS*, *PPP2R1A, PIK3CA, ARID1A*, *B-*Raf Proto-Oncogene, Serine/Threonine Kinase (*BRAF)*, NRAS Proto-Oncogene, GTPase (*NRAS)*, HRAS Proto-Oncogene, GTPase (*HRAS)*, Extracellular Signal-Regulated Kinase 1 (*ERK1)* and *ERK2* and *PTEN* in 101 ovarian endometriosis samples found only four mutations in three lesions, including a *KRAS* p.G12V, *PPP2R1A* p.S256F mutation, and two *ARID1A* nonsense mutations (p.Q403 and p.G1926) (Zou *et al.*, 2018a).

Studies targeting the mutant alleles of *KRAS* p.G12V in 26 endometriomas identified 10 mutations, with suggestions that these mutations were associated with inflammation and intratumor heterogeneity (Yachida *et al.*, 2021). Another study on *KRAS* mutations on codons 12 and 13 in 12 OMA samples found no mutations (Kim *et al.*, 2018). A targeted approach has also been used to investigate Transcription regulating factor 1 (*TRERF1*) (2/92, 2.2%) (Cao *et al.*, 2018), CCCTC-binding factor (*CTCF*) (2/92, 2.2%), and Myosin Heavy Chain 8 (*MYH8)* mutations (2/152) (Lou *et al.*, 2020). Examination of the entire coding sequence of Caspase Recruitment Domain Family member 10 (*CARD10)* and *CARD11* in 101 patients with ovarian endometriosis identified four novel somatic mutations, two in-frame deletions of *CARD10* and two heterozygous missense mutations in *CARD11* (Zou *et al.*, 2018b). A relationship between endometrioma mutations and clinical outcomes is still lacking. However, there is some evidence that KRAS mutations and somatic PTEN loss are associated with disease severity (Orr *et al.*, 2023; Tucker *et al.*, 2025). KRAS mutations and PTEN loss are correlated with more severe anatomical subtypes such as DIE and OMA.

### Superficial peritoneal endometriosis

The mutational profile of SUP is yet to be investigated as thoroughly as either OMA or DIE lesions. One investigation on 40 iatrogenic lesions excised from various locations, including the abdominal wall and recto-uterine pouch, performed in parallel with DIE lesions, identified mutations in four patients, including *KRAS* (2/40), *PIK3CA* (1/40), and *ERRB2* (1/40), although with a rate of mutations less than that observed in DIE lesions (Lac *et al.*, 2019). A study on rare cases of endometriosis-associated intestinal tumours in two patients using whole exome sequencing identified frameshift mutations in *ARID1A*, *PTEN*, and *p53* (Kurose *et al.*, 2021). Another study that included 18 SUP lesions from a Korean population found no mutations in codon 12 or 13 of *KRAS* (Kim *et al.*, 2018).

### Comparison between endometriosis subtypes

Common mutations that occur in different lesions are of particular interest as they suggest a shared precursor mutation and may provide insight into disease pathogenesis. As such, there have been attempts to compare rates and identity of mutations across endometriosis subtypes within the same patient. In one case, samples were collected from the right and left ovary of the same patient and investigation of their mutational status found that one lesion harboured mutations in *KRAS*, whereas the other lesion contained a mutation in *PTEN*, suggesting that either these lesions were not seeded from the same precursor cell, or that they had developed differently over time (Suda *et al.*, 2018).

Differences in the rate of mutations across subtypes have also been observed. Analysis of *KRAS* codon 12 mutations indicated variations in anatomical subtypes with superficial-only lesions (35.1%), showing the lowest proportion of mutations when compared to either OMA only, DIE only (60.6%), or mixed anatomic subtypes (60.6%), supporting an association of increased mutations with more severe or progressed lesions (Orr *et al.*, 2023). In 59 lesion samples, hotspot mutations were observed in 27 samples (45.8%) with the most common being *PIK3CA*, followed by *KRAS* and *CTNNB1*: a trend that was consistent in all lesion subtypes, although with OMA showing the highest percentage of mutations (Praetorius *et al.*, 2022). Significantly, these authors reported evidence of identical mutations in different lesions within the same patient, suggesting a common ancestor cell (Praetorius *et al.*, 2022), supporting a shared pathogenesis. *KRAS* G12D was the most likely mutation to become clonal (Orr *et al.*, 2023).

### Adenomyosis

Adenomyosis is characterized by endometrial-like epithelium and stroma invading the myometrium, often leading to uterine enlargement, pain, and prolonged menstrual bleeding (Buggio *et al.*, 2021). Mutational profiling of epithelial cells in adenomyosis identified 134 unique synonymous and non-synonymous single-nucleotide polymorphisms (SNPs) in 31 out of 51 adenomyosis patients, with a mean of 2.6 mutations per individual with a low variant allele frequency mean of 4.8% (Inoue *et al.*, 2019), similar to endometriosis (Table 1). *KRAS* mutations were found in 37.1% of the cases, most commonly in p.G12, and PIK3CA mutations were also confirmed (Inoue *et al.*, 2019). Importantly, the presence of *KRAS* mutations was also observed in surrounding normal epithelial cells, supporting the theory that the acquisition of *KRAS* mutations enhances the invasiveness and proliferative capacity. Sequencing in 17 patients also identified *KRAS* and *ARID1A* as the most commonly mutated genes (Chao *et al.*, 2023). More recently, an examination of 16 adenomyosis samples identified mutations in 81 different genes, although only one of these, *PIK3CA*, appeared in more than one sample (two samples) with a higher mutation per sample than in endometrial or endometriosis samples (Li *et al.*, 2021). It has been proposed that the association of adenomyosis with parity might be mediated through an increase in mutations in normal endometrium induced by physical stressors, including uterine contractions and vaginal delivery, which lead to increased cellular proliferation (Inoue *et al.*, 2020).

### Potential biological and functional consequences of endometrial somatic mutations

**Figure 2.**
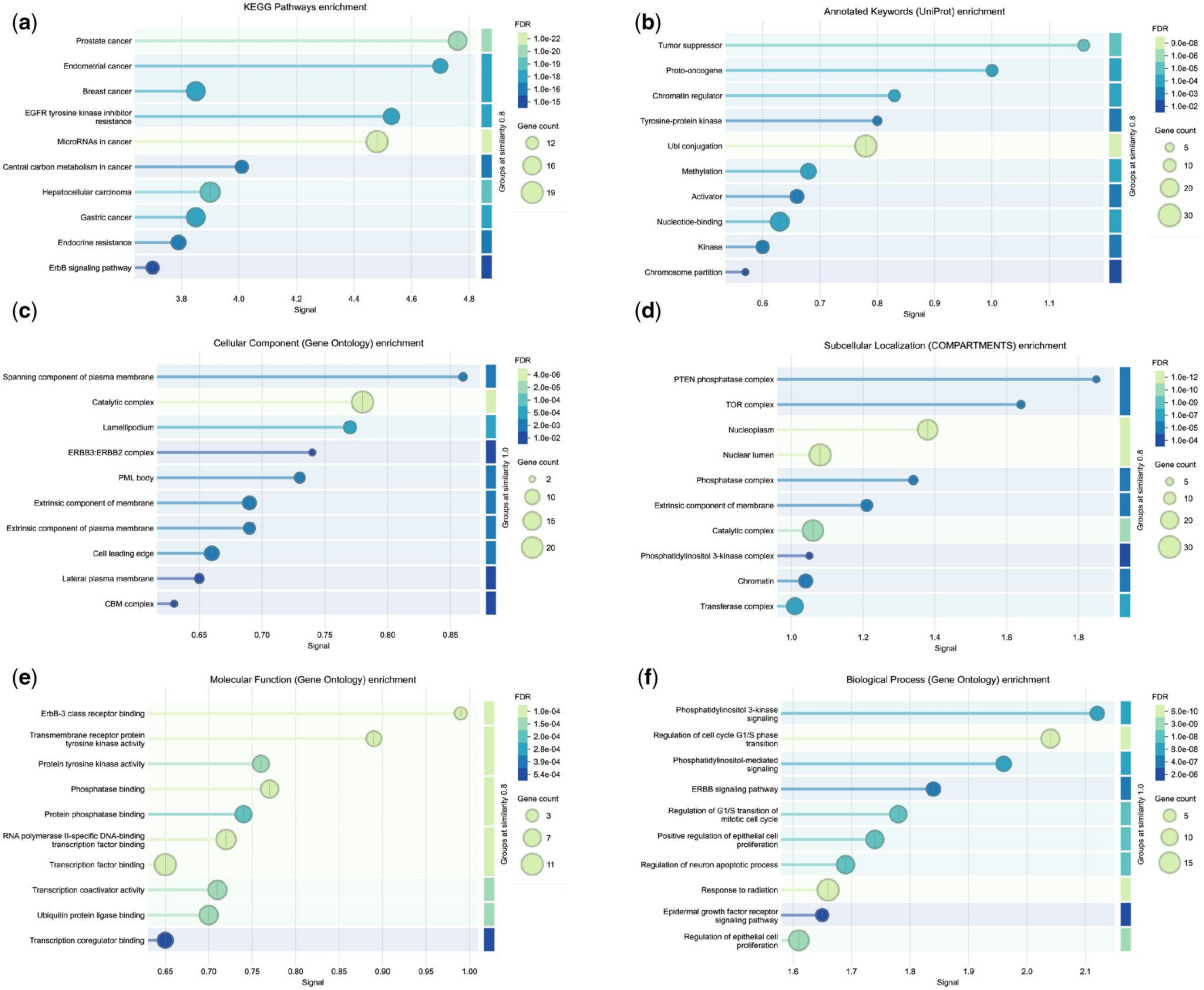
**Functional enrichment analysis of genes carrying somatic mutations in endometrial tissue**. (**a**) KEGG pathway enrichment analysis identified endometrial cancer and prostate cancer pathways as the most significantly enriched pathways (**b**) Functional annotation using UniProt identified enrichment of genes classified as tumour suppressors and proto-oncogenes. (**c**) Subcellular localization analysis revealed that many encoded proteins are associated with the plasma membrane. (**d**) Protein complex enrichment analysis identified significant associations with the PTEN signaling complex and the mTOR complex. (**e**) Gene ontology enrichment analyses of molecular function showed significant over-representation of pathways related to ErbB signaling. (**f**) Biological process enrichment analyses identified signaling pathways related to phosphatidylinositol kinase activity.

An analysis of the canonical function of all genes associated with somatic mutations (Table 1) suggested roles in cell proliferation, growth, migration, transcriptional and genomic dysregulation, immune or hormone signaling changes, and telomerase activity changes. To further investigate the potential biological consequences of these mutations, a combined analysis using the STRING database (von Mering *et al.*, 2003) was performed. All genes listed in Table 1 were combined, duplicates were removed, and the resulting gene set was interrogated against the human database to perform functional enrichment and network analysis. Pathway enrichment using KEGG identified endometrial cancer and prostate cancer pathways as the most significantly enriched pathways (Fig. 2A; Supplementary Table S1). Annotation using UniProt further indicated enrichment of genes classified as tumour suppressors and proto-oncogenes (Fig. 2B; Supplementary Table S2). Subcellular localization analysis revealed that many of the encoded proteins are associated with components of the plasma membrane (Fig. 2C; Supplementary Table S3). Protein complex enrichment highlighted associations with both the PTEN signaling complex and the mTOR complex (Fig. 2D; Supplementary Table S4). Consistent with these findings, molecular function and biological process enrichment analyses identified signaling pathways related to ErbB signaling and phosphatidylinositol kinase activity as significantly over-represented (Fig. 2E and F; Supplementary Tables S5 and S6). This analysis therefore suggests that somatic mutations in the endometrium converge on signaling pathways and may have critical impacts on the regulation of epithelial growth, survival and cellular homeostasis, particularly within the PI3K–PTEN–mTOR axis.

## *In vitro* models to study mutations in endometrial pathologies

Characterizing the consequences of somatic mutations in well-defined epithelial cell models will help us understand the contribution of individual mutations and the potential to target treatments towards them. Various laboratory models have been used in the past, but have been limited by their inability to recapitulate the complex endometrial environment and by the resistance of these cells to long-term culture. The development of increasingly complex patient-derived *in vitro* models is now providing the necessary tools to understand the contribution of mutations to endometrial pathologies and to trial different therapeutics for individual responses.

### Immortalized epithelial cells

**Figure 3.**
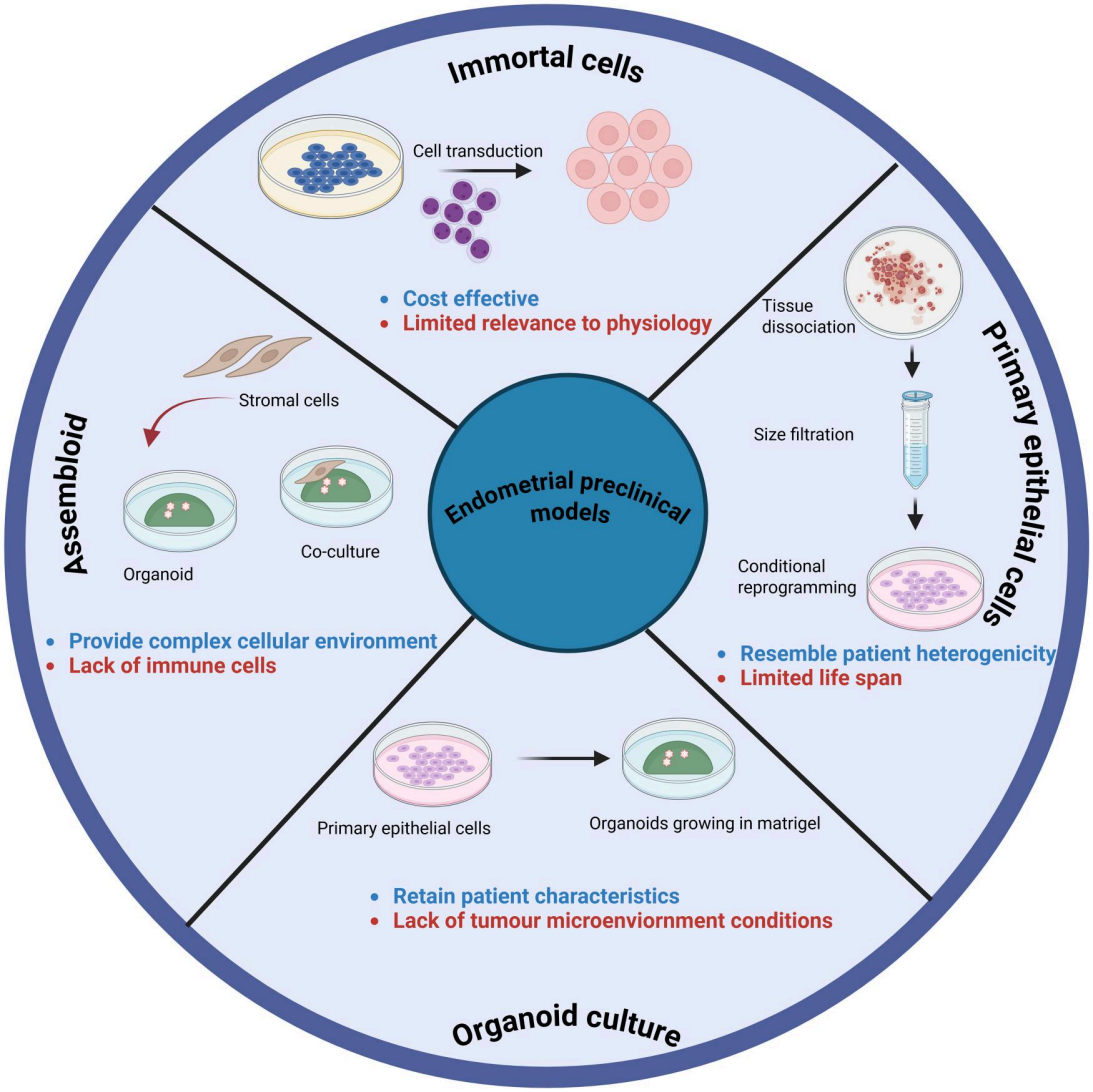
***In vitro* models to study somatic mutations in endometrial pathologies**. Endometrial models include immortalized cell lines, primary human epithelial cells, organoids and assembloids. Immortal cells are derived from epithelial tissues, genetically modified to retain proliferative capacity. They are limited by genetic drift and an inability to capture patient heterogeneity. Primary human endometrial epithelial cells derived from resected human tissue can be cultured as monolayers under specific conditions. They retain the individual patient character; however, they are limited by a finite life span, and they lack cellular polarity and three-dimensional structure. Organoids are patient-derived epithelial cells grown from epithelial fragments that generate multiple cell types of the epithelial lineage to create polarized cells that maintain three-dimensional gland-like structures. Organoids better recapitulate the *in vivo* environment as they contain multiple cell states but still lack a tumour microenvironment. Assembloids are organoid co-cultures and endometrial stromal cells that mimic the microenvironment. While they are an improvement, they still lack vascularization and immune components. Created in BioRender. Subramaniam, S. (2026) https://BioRender.com/hjxfzc8.

Immortalized endometrial epithelial cell models were widely used in the past (Table 2) (Fig. 3). The introduction of telomerase reverse transcriptase protein (TERT) into proliferative endometrial epithelial cells overcomes replicative senescence (Boccellino *et al.*, 2012) and provides a model that can easily be cultured for long periods. Many examples have been produced and manipulated in the past. The hEM3 cell line was established through immortalization and clonal selection and retains the natural characteristics of endometrial epithelium (Park *et al.*, 2021).

**Table 2.** Summary of endometrial pre-clinical models and their key features.

Endometrial pre-clinical models	Advantages	Disadvantages	Major applications
Immortalized epithelial cells	Easy to culture and expand Cost effective Infinite growth	Limited by their inability to recapitulate the complex and dynamic endometrial tumour microenvironment Lack of cellular heterogeneity Limited cell interactions Genetic drift in long periods of culture	Drug screening Functional assays
Primary epithelial cells	Retain the individual characteristics of the patient	Limited lifespan Slow growth rate Represent only a single differentiated state of the cell Do not recapitulate the complex and dynamic endometrial microenvironment	Functional assays Assays to identify invitro effects Drug response Molecular and genetic studies
Organoids	Recapitulates the properties and features of in vivo tissue Genetically stable Long term experiments possible	Matrigel does not mimic natural endometrial extra cellular matrix Lack of immune and stromal cell interaction	Mutational and genetic studies Drug screening studies and personalized medicine High throughput screening studies
Organoid-co-culture	Mimic dynamic endometrial microenvironment Study of cell–cell interactions	Time consuming experimental setup Limited complexity compared to endometrium	High-throughput drug screening studies Mutational and genetic studies Tool for precision therapy

Immortalized endometrial epithelial cells have been used to investigate the consequences of *KRAS* and *PIK3CA* mutations (Hossain *et al.*, 2021). HMOsisEC10 (Wild-type), *KRAS*-mutant, and *PIK3CA*-mutant cell lines were established from surface epithelial tissue of ovarian endometriosis by the triple expression of an active *CDK4* mutant (CDK4^R24G^), *cyclin D1*, and *hTERT*. The mutant cell lines exhibited a higher rate of cell proliferation, invasion, and migration as well as resistance to premature senescence. Neither of the mutant cell lines developed colonies in the soft agar assay, strengthening the theory that multiple genetic mutations are essential for a benign-to-malignant transformation to occur.

While useful, immortalized cell lines have limitations, particularly for studying the consequences of mutations. They are limited by patient heterogeneity and by their inability to recapitulate the complex and dynamic endometrial environment or early developmental cell states. They also rely on the continued expression of exogenous genes, which can induce genomic instability, making it difficult to be sure the observed effects are due to the mutation of interest and not the consequence of genetic drift that occurs over long periods of culture. It is for these reasons that more complex *in vitro* models are being sought for studying the genetic contribution towards endometrial epithelial cell pathologies.

### Primary epithelial cells

Primary endometrial epithelial cells derived from resected human tissue can be cultured as monolayers (Chen and Roan, 2015) to avoid some of the limitations associated with immortalized cell lines (Kyo *et al.*, 2003). They can also retain the individual characteristics of the patient, an important feature when studying heterogeneous diseases. They are, however, limited by a finite life span (Table 2) and several approaches have been used to overcome this limitation (Awatade *et al.*, 2018). Conditional reprogrammed epithelial cells (CRC) (Liu *et al.*, 2012; Suprynowicz *et al.*, 2012) are established by maintaining epithelial cells with irradiated mouse fibroblast feeder cells (Wu *et al.*, 2020) in specialized media that inhibits Rho-associated protein kinase (ROCK). This method stimulates the continuous doubling of epithelial cells without compromising the characteristic epithelial cell morphology and genome stability.

Similar to immortalized cell lines, there are limitations for these conditionally reprogrammed primary cells. Cells grown as monolayers represent only a single differentiated state of the cell and they lack the critical contributions of the surrounding microenvironment. Studies have reported that epithelial cells fail to reproduce the complex and dynamic environments of *in vivo* tissues and may not be responsive after a few passages (Iruela-Arispe *et al.*, 1999; Hibaoui and Feki, 2020).

### Human endometrial organoids

Organoids are patient-derived *in vitro* models generated from adult stem cells that are cultured in 3D to recapitulate the properties and features of the *in vivo* tissue (Table 2, Fig. 3). Single-cell analysis of estrogen-treated organoids identified a mixture of epithelial cells, including proliferative, ciliated, unciliated, and stem-cell types, with organoids treated with estrogen, progesterone, and cAMP, revealing a secretory cell population (Fitzgerald *et al.*, 2019). The number of ciliated cells increased with estrogen treatment, and similar results were found *in vivo*, supporting estrogen regulation of cilia formation.

A number of studies are beginning to show that organoids are a reliable model to recapitulate endometrial pathologies (Boretto *et al.*, 2019; Marr *et al.*, 2025). However, limitations of these models still exist. Reproducibility is limited by the ill-defined contents of commonly used hydrogels for 3D growth and the lack of non-epithelial cells such as mesenchymal, endothelial or immune cells in the models, limiting their resemblance to the *in vivo* environment.

Organoids offer great potential for studying the influence of somatic mutations in multipotent, clonally expanding cells. Endometrial mutations in *FBXW7*, *ARID1A*, and *PTEN* in primary microsatellite instability tumours were retained in organoids with serial passaging, and 21 of the most frequently mutated genes in endometrial cancer tumours were identified in organoids derived from this tissue (Boretto *et al.*, 2019). This result suggests that organoids closely resemble the tumour tissue at the genetic level and represent a significant advance in the ability to study the acquisition and consequence of mutations in endometrial epithelial cells.

### Multi-lineage organoid cultures

Assembloids are organoids that have been co-cultured with stromal cells to address some of the limitations noted above (Rawlings *et al.*, 2021) (Table 2, Fig. 3). Co-culturing of epithelial and stromal cells on collagen scaffolds was also confirmed with the EPCAM^+^ epithelial and EPCAM^-^ stromal cells (Abbas *et al.*, 2020). A recently published paper illustrated the importance of multicellular organoid co-culture models and the introduction of defined matrices to study their cellular interactions (Gnecco *et al.*, 2023). These studies demonstrated that co-culture models generate significant insights into the molecular dynamics in the development of endometrium and reproductive disorders. There remain challenges, however, in strengthening these models further, as they currently lack blood vessels or immune cells. Additionally, due to the dynamic nature of the endometrium, synchronization of the relative developmental stage of each cell type could have significant impacts on their interaction and cellular behaviour.

### Genetic manipulation of epithelial *in vitro* models

Gene editing of *in vitro* models is used to study pathology and mutations and to validate drug targets. In the case of organoids established from progenitor cells, there is also the potential to assess mutations on cellular differentiation. Genomic editing of endometrial epithelial *in vitro* models has been limited to date, potentially due to the difficulties in establishing epithelial cell models, but also through the need to manipulate cells in their stem cell state and outside the supporting Matrigel to effectively achieve transduction.

Genetic manipulation has however been performed in intestinal organoids (Ringel *et al.*, 2020), colon cancer (Boretto *et al.*, 2024), and cystic fibrosis-derived intestinal organoids (Schwank *et al.*, 2013). Stable adult stem cell knock-in organoids have also been generated for colon cancer (Cortina *et al.*, 2017; Shimokawa *et al.*, 2017). The evolution of the complex *in vitro* models of the endometrial environment, coupled with our rapidly expanding ability to create genetic perturbations, is creating the potential to elucidate the consequences of genetic variants in endometrial epithelial cells.

## Conclusion

The epithelial cells of the endometrium provide critical roles as barrier cells and for the production of histotrophic nutrients. They are characterized by high turnover and rapid replacement. This regenerative capacity is driven by progenitor cells residing in horizontal glandular structures in the basalis that produce vertical glands during the menstrual cycle. Somatic mutations, including known cancer driver mutations, are frequent in these endometrial epithelial cells, but we are yet to understand their functional consequences and their likely significant contributions to aberrant development. Endometriosis and adenomyosis are chronic endometrial disorders that have previously been associated with mutations within the endometrial epithelium.

The somatic mutations found frequently within endometrial epithelial cells are often well-studied cancer driver mutations, such as those affecting KRAS, PIK3CA, and ARID1A. The functional consequences of these mutations remain poorly understood, and their potential contributions to the initiation of pathological conditions have yet to be fully elucidated. The cataloguing of these mutations and a subsequent STRING analysis has shown that there is the potential for these somatic mutations to impact genes involved in growth factor signaling and PI3K-mediated cellular regulation: pathways that are central to endometrial. These functions are often associated with both benign and malignant endometrial transformation.

Although genetic alterations are often observed in cancer-associated mutations in epithelial cells, it does not necessarily lead to malignant transformation. Several factors could contribute to this, including clonal competition, the influence of the tissue microenvironment, immune surveillance, and the requirement for additional genetic or epigenetic alterations for tumour initiation. Furthermore, the current genomic datasets have limitations for studying mutations. Many studies rely on conventional next-generation sequencing and bulk sequencing approaches, which have limited sensitivity for detecting low-frequency or rare variants and do not account for cellular heterogeneity. Overcoming these limitations requires more comprehensive methods to better understand the functional consequences of somatic mutations and their roles in endometrial pathology.

New technologies such as advanced 3D models, potentially coupled with genetic manipulation, now offer the opportunity to study the functional impact and consequences of these somatic alterations. These approaches can not only help clarify how mutations influence epithelial behaviour but also facilitate the identification of compounds that could be targeted to treat individual patients based on their mutational profiles, thereby enabling personalized therapeutic strategies. This highlights the need for a more comprehensive catalogue of somatic mutations involved in endometrial pathologies, as well as functional studies to identify their biological consequences. Such knowledge will play a key role in our understanding of the mechanisms underlying endometrial diseases and in advancing the development of precision medicine approaches.

## Supplementary Material

gaag027_Supplementary_Data

## Data Availability

No new data were generated in support of this article.
