# Supplementary material for: Somatic mutations in endometrial epithelium: biological insights and emerging in vitro models
Source: Mol Hum Reprod. 2026 May 9;32(2):gaag027. doi: 10.1093/molehr/gaag027 (PMC13275306; doi:10.1093/molehr/gaag027)
Supplement: gaag027_Supplementary_Data [file Review_comments_and_author_responses_MHR-25-0360.pdf › Review_comments_and_author_responses_MHR-25-0360.pdf]

Reviews and authors' responses for manuscript 'Somatic mutations in endometrial epithelium: biological insights and emerging *in vitro* models' by Sugarniya Subramaniam *et al*.

Original submission: MHR-25-0360

Reviewer 1
This manuscript provides a concise review of the current understanding of somatic mutations in the human endometrium and their potential relevance to conditions such as endometriosis and adenomyosis. The topic is timely, and the authors effectively summarize existing research regarding epithelial mutational burden, possible mechanisms of mutation acquisition, and the challenges posed by the lack of robust in vitro tracing systems. While the review is generally clear and well organized, several areas would benefit from further refinement.

In particular, this manuscript could provide a more systematic framework for evaluating the strength of evidence supporting specific mutation–phenotype relationships, as some sections rely heavily on descriptive summaries. Also, adding a brief annotation of the gene function in the table 1 could help interpret the summary.

Additionally, expanding the summary and discussion section could improve the readability and further strengthen the significance of the study.

Finally, discussion to unresolved controversies and the limitations of current genomic datasets would help contextualize the conclusions (i.e. oncogenic mutations acquired in the epithelial cells does not necessitate the cancer formation in the female reproductive tract).

Some minor comments:
The statement from Line 54 line needs references to support the claim.
The statement from Line 155-156 is very broad, please add discussion/evidence to give more content.

Some additional background summary/introduction of the pathologies discussed in this review might be appropriate for the structural integrity of this manuscript.

Reviewer 2
This review summarizes the recent advancement about the development of endometrial epithelium during physiological and pathological conditions and emphasizes on the mutational landscape. It is an excellent review for the general field.
I have one minor comment. The title of the review is about endometrium. But the paper just starts with the introduction of endometrial epithelium. It has been reported that the epithelial compartment has more mutations. But for readers in the general field, I think a brief explanation about why focus on epithelium would be more logically sounding.

Associate Editor
The review by Subramaniam provides an up-to-date discussion of the state of the literature regarding the prevalence of somatic cell mutations in the endometrial epithelium and their

potential consequence to endometrial pathologies. The manuscript offers detailed reviews of recent primary literature about the presence of somatic mutations, with a focus on their implications to endometriosis and adenomyosis. While the authors provide a comprehensive introduction to endometrial epithelial subtypes, their development, roles and functions, it might be helpful to also briefly describe stromal cells and why the focus of the review is the epithelium. The reviewers agree that this is a well written and comprehensive review and offer minor edits that would improve the readability of the review. These suggestions include adding details regarding the functional consequence of the endometrial mutations to both table 1 and the text. It is also suggested that the authors edit the manuscript title to more closely reflect the content of the review; as stated, the title implies that this a study rather than a review of the literature and that the focus could be on mutations found in both endometrial epithelium and stroma. After reading the manuscript, I have also offered other minor corrections noted below.
Line 132-136: Although it is implied, the authors should clarify whether the multiple cancer associated genes found in the 11 endometrial glands were mutated.
There is a minor grammatical error in Line 369: Organoids have been co-cultured with stromal cell and in the summary section, Line 401: …are frequent in these endometrial epithelial [cells?] but we are yet to understand their functional…

Editor-in Chief

The reviewing team is unanimous in considering that this is a well-written comprehensive review on a timely and deserving topic, namely, the occurrence and prevalence of somatic mutations in the epithelial compartment of the endometrium. In addition, the Authors also summarized the models that are available to study those mutations e.g. organoids. The manuscript can be considered for publication pending minor revision. It would be redundant to repeat what the Reviewers noted and the Associate Editor brought to the point. It has been noted that the title could be edited to more closely reflect the content of the manuscript, and that Table 1 could include an extra column to name the main canonical function of the mutated genes. Our Reviewers and Associate Editor made also other remarks, which the Authors should take to heart. In addition to the sensible comments made by Reviewer and Associate Editor, the Editor in Chief would like to note the following. Table 1 features more than 100 genes. This is probably a critical mass to run a meaningful gene network and/or gene ontology analysis. So, the EiC loaded the list of genes to STRING. The application returned a network and an analysis, for example:

top GO BP term is 'ERBB signaling pathway'
top KEGG pathway is 'endometrial cancer'
top keyword in UniProt is ‘tumor suppressor’
top protein domain is ‘Ras subfamily of RAS small GTPases’
and much more…

This seems like a wealth of information that could be useful to the Authors in refining their review. One Reviewer indeed suggested to expand the summary and discussion section, and the STRING analysis could offer an opportunity to address the Reviewer’s suggestion. MHR is a journal with an emphasis on molecular processes and mechanisms. It would be nice if the Authors could enrich their already excellent review with considerations and insights based on e.g. gene ontology analysis. It appears that the genes in Table 1 have some things in common.

We think that the review has potential to be highly appreciated by the readership, particularly after the improvements that could be made through a revision. The Authors are in a strong position to revise. Finally, please can the minor formatting issues identified by the Editorial Office (below) also be addressed. We look forward to receiving the R1 of this manuscript.

Authors' Response to Decision Letter for MHR-25-0360

We sincerely thank the editor and reviewers for their careful evaluation of our manuscript and for the valuable comments and suggestions. We have revised the manuscript accordingly and addressed all comments in detail below. All changes have been marked in red in the revised manuscript.

Editor-in-Chief

The reviewing team is unanimous in considering that this is a well-written comprehensive review on a timely and deserving topic, namely, the occurrence and prevalence of somatic mutations in the epithelial compartment of the endometrium. In addition, the Authors also summarized the models that are available to study those mutations e.g. organoids. The manuscript can be considered for publication pending minor revision. It would be redundant to repeat what the Reviewers noted and the Associate Editor brought to the point. It has been noted that the title could be edited to more closely reflect the content of the manuscript, and that Table 1 could include an extra column to name the main canonical function of the mutated genes. Our Reviewers and Associate Editor made also other remarks, which the Authors should take to heart. In addition to the sensible comments made by Reviewer and Associate Editor, the Editor in Chief would like to note the following. Table 1 features more than 100 genes. This is probably a critical mass to run a meaningful gene network and/or gene ontology analysis. So the EiC loaded the list of genes to STRING. The application returned a network and an analysis, for example:

top GO BP term is 'ERBB signaling pathway'
top KEGG pathway is 'endometrial cancer'
top keyword in UniProt is 'tumor suppressor'
top protein domain is 'Ras subfamily of RAS small GTPases'
and much more…

This seems like a wealth of information that could be useful to the Authors in refining their review. One Reviewer indeed suggested to expand the summary and discussion section, and the STRING analysis could offer an opportunity to address the Reviewer's suggestion. MHR is a journal with an emphasis on molecular processes and mechanisms. It would be nice if the Authors could enrich their already excellent review with considerations and insights based on e.g. gene ontology analysis. It appears that the genes in Table 1 have some things in common.
We think that the review has potential to be highly appreciated by the readership, particularly after the improvements that could be made through a revision. The Authors are in a strong position to revise. We look forward to receiving the R1 of this manuscript.

Response: We sincerely thank the Editor-in-Chief, Associate Editor, and Reviewers for their positive evaluation of our manuscript. We greatly appreciate the constructive suggestions,

which have helped us improve the clarity of the review. We have carefully revised the manuscript in response to all comments.

- Page 1, lines 1-2: We have revised the title to better reflect the scope and content of the manuscript. The title has been changed to “Somatic Mutations in Endometrial Epithelium: Biological Insights and Emerging In Vitro Models”
- Table 1: has been updated to include an additional column describing the main canonical function of the mutated genes.
- Following the EiC suggestion, we performed a gene network and gene ontology analysis using STRING based on the genes listed in Table 1. The results of this analysis revealed enrichment in several biological processes and pathways, including the ERBB signaling pathway, endometrial cancer–related pathways, and genes associated with tumor suppressor functions and Ras-related signaling. These findings have now been incorporated into the manuscript in a new section entitled “Potential biological and functional consequences of endometrial somatic mutations”
- We have expanded the summary and discussion sections

We hope that the revisions have further improved the manuscript and we thank the Editor and Reviewers again for their valuable feedback.

Associate Editor

The review by Subramaniam provides an up-to-date discussion of the state of the literature regarding the prevalence of somatic cell mutations in the endometrial epithelium and their potential consequence to endometrial pathologies. The manuscript offers detailed reviews of recent primary literature about the presence of somatic mutations, with a focus on their implications to endometriosis and adenomyosis. While the authors provide a comprehensive introduction to endometrial epithelial subtypes, their development, roles and functions, it might be helpful to also briefly describe stromal cells and why the focus of the review is the epithelium.

Response: We thank the Associate Editor for this suggestion. Previous studies have shown there is a greater rate of mutations in the epithelial cells compared to the stromal cells. We have added a brief paragraph in the main text, **Page 3, Lines 55-61** explaining the rationale for focusing on the endometrial epithelium rather than the stromal cells.

“These mutations are largely confined to epithelial cells. In micro-dissected endometriotic lesions somatic mutations were significantly enriched in the epithelium and not in the stromal component (Noe et al. 2018). Whole‐exome and targeted sequencing studies of deep infiltrating endometriosis (DIE) also reported that known cancer driver mutations were limited to the epithelial cells, with no detectable mutations in the stromal cells of the same lesions (Anglesio et al. 2017).. Together, these findings support epithelial cells as the primary carriers of somatic alterations in endometriosis, whereas stromal cells do not show the same frequency of mutations.”

The reviewers agree that this is a well written and comprehensive review and offer minor edits that would improve the readability of the review. These suggestions include adding details regarding the functional consequence of the endometrial mutations to both table 1 and the text.

Response: Table 1: has been updated to include an additional column describing the main canonical function of the mutated genes. Additionally, the text has been updated to highlight the functional consequences of these mutations on the genes, Page 11, Lines 285-288. As up to 100 genes have been described and the information is available in the text we have endeavoured to keep this brief.

"Potential biological and functional consequences of endometrial somatic mutations
The canonical function of all genes associated with somatic mutations was examined (Table 1) suggesting roles in cell proliferation, growth, migration, transcriptional and genomic dysregulation, immune or hormone signaling changes, and telomerase activity changes. To further investigate a potential biological consequence of these mutations a combined analysis using the STRING database (von Mering et al. 2003) was performed. All genes listed in ***Table 1*** *were combined, duplicates removed, and the resulting gene set interrogated against the human database to perform functional enrichment and network analysis. Pathway enrichment using KEGG identified endometrial cancer and prostate cancer pathways as the most significantly enriched pathways (****Figure 2a; Supplementary Table 1****). Annotation using UniProt further indicated enrichment of genes classified as tumor suppressors and proto-oncogenes (****Figure 2b; Supplementary Table 2****). Subcellular localization analysis revealed that many of the encoded proteins are associated with components of the plasma membrane (****Figure 2c; Supplementary Table 3****). Protein complex enrichment highlighted associations with both the PTEN signaling complex and the mTOR complex (****Figure 2d; Supplementary Table 4****). Consistent with these findings, molecular function and biological process enrichment analyses identified signaling pathways related to ErbB signaling and phosphatidylinositol kinase activity as significantly over-represented (****Figure 2e–f; Supplementary Tables 5–6****). From this analysis it would suggest somatic mutations in the endometrium converge on signaling pathways and may have critical impacts on the regulation of epithelial growth, survival, and cellular homeostasis, particularly within PI3K–PTEN–mTOR axis."*

It is also suggested that the authors edit the manuscript title to more closely reflect the content of the review; as stated, the title implies that this a study rather than a review of the literature and that the focus could be on mutations found in both endometrial epithelium and stroma.

Response: Page 1, lines 1-2: We have revised the title to better reflect the scope and content of the manuscript. The title has been changed to "*Somatic Mutations in Endometrial Epithelium: Biological Insights and Emerging In Vitro Models*"

After reading the manuscript, I have also offered other minor corrections noted below.
Line 132-136: Although it is implied, the authors should clarify whether the multiple cancer associated genes found in the 11 endometrial glands were mutated.

Response: We have revised this section to explicitly state which cancer-associated genes were found to carry mutations in the sampled glands **Page 6, lines 133-136.**

"Whole-exome and targeted sequencing of 11 endometrial glands identified mutations in multiple cancer-associated genes, including phosphatidylinositol -4,5- Bisphosphate 3-Kinase Catalytic Subunit Alpha (PIK3CA), Kirsten Rat Sarcoma Viral Oncogene Homolog

(KRAS), Rho GTPase Activating protein 35 (ARHGAP35) and Phosphoinositide-3Kinase Regulatory Subunit 1 (PIK3RA) (Suda et al. 2018).”

There is a minor grammatical error in Line 369: Organoids have been co-cultured with stromal cell and in the summary section

Response: This grammatical error has been corrected in the revised manuscript. Page 14, line 379

“Organoids have been co-cultured with stromal cells to address some of the limitations noted above (Rawlings et al. 2021) (Table 2) (Figure 3).”

Line 401: ...are frequent in these endometrial epithelial [cells?] but we are yet to understand their functional…

Response: This grammatical error has been corrected in the revised manuscript. Page 15, line 410-412

“Somatic mutations, including known cancer driver mutations, are frequent in these endometrial epithelial cells but we are yet to understand their functional consequences and their likely significant contributions to aberrant development.”

Reviewer: 1

This manuscript could provide a more systematic framework for evaluating the strength of evidence supporting specific mutation–phenotype relationships, as some sections rely heavily on descriptive summaries.

Response: We thank the reviewer for this useful suggestion. To address this point, we have revised the table 1 to include the canonical function of all genes identified as requested by the EiC and associate editor. We have also included some additional text to provide a clearer framework for evaluating the evidence linking somatic mutations with specific endometrial phenotypes **Page 11, Lines 286-288**.

“The canonical function of all genes associated with somatic mutations was examined (Table 1) suggesting roles in cell proliferation, growth, migration, transcriptional and genomic dysregulation, immune or hormone signaling changes, and telomerase activity changes.”

Adding a brief annotation of the gene function in Table 1 could help interpret the summary.

Response: We have revised Table 1 to include a brief description of the canonical function of each gene to improve clarity and facilitate interpretation for readers.

Expanding the summary and discussion section could improve readability and strengthen the significance of the study.

Response: The summary section has been expanded to better synthesize the key findings of the review, highlight the implications of somatic mutations in the human endometrium, and highlight the important directions for future research **Pages 15-16, lines 406-443**

"Summary
The epithelial cells of the endometrium provide critical roles as barrier cells and for production of histotrophic nutrients. They are characterized by high turnover and rapid replacement. This regenerative capacity is driven by progenitor cells residing in horizontal glandular structures in the basalis that produce vertical glands during the menstrual cycle. Somatic mutations, including known cancer driver mutations, are frequent in these endometrial epithelial cells but we are yet to understand their functional consequences and their likely significant contributions to aberrant development. Endometriosis and adenomyosis are chronic endometrial disorders that have previously been associated with mutations within the endometrial epithelium.

The somatic mutations found frequently within endometrial epithelial cells are often well studied cancer driver mutations such as those affecting KRAS, PIK3CA and ARID1A. The functional consequences of these mutations remain poorly understood and their potential contributions to the initiation of pathological conditions have yet to be fully elucidated. Through the cataloguing of these mutations and a subsequent STRING analysis there is the potential for these somatic mutations to impact genes involved in growth factor signaling and PI3K-mediated cellular regulation, pathways that are central to endometrial. These functions are often associated with both benign and malignant endometrial transformation.

Although genetic alterations are often observed in cancer-associated mutations in epithelial cells it does not necessarily lead to malignant transformation. Several factors could contribute to this, including clonal competition, the influence of the tissue microenvironment, immune surveillance, and the requirement for additional genetic or epigenetic alterations for tumour initiation. Furthermore, current genomic datasets have limitations in studying mutations. Many studies rely on conventional next-generation sequencing and bulk sequencing approaches, which have limited sensitivity for detecting low-frequency or rare variants and do not account for cellular heterogeneity. Overcoming these limitations requires more comprehensive methods to better understand the functional consequences of somatic mutations and their roles in endometrial pathology.

New technologies such as advanced 3D models, potentially coupled with genetic manipulation now offer the opportunity to study the functional impact and consequences of these somatic alterations. These approaches can not only help clarify how mutations influence epithelial behaviour but also facilitate the identification of compounds that could be targeted to treat individual patients based on their mutational profiles, thereby enabling personalised therapeutic strategies. This highlights the need for a more comprehensive catalogue of somatic mutations involved in endometrial pathologies, as well as functional studies to identify their biological consequences. Such knowledge will play a key role in understanding the mechanisms underlying endometrial diseases and in advancing the development of precision medicine approaches."

Minor comment: Discussion of unresolved controversies and the limitations of current genomic datasets would help contextualize the conclusions (i.e., oncogenic mutations acquired in epithelial cells do not necessarily lead to cancer formation).

Response: We thank the reviewer for this important point. We have expanded the summary to address current controversies and limitations in the field. We also discuss why the presence of oncogenic mutations does not necessarily lead to malignant transformation. These additions can be found in the summary section **Pages 15-16, lines 406-443**

See Summary above for Reviewer 1: Comment 3.

Minor comment: The statement from Line 54 needs references to support the claim.

Response: Thank you for noting this. Appropriate references have now been added to support this statement Page 3, lines 55

"During regeneration, endometrial epithelial cells acquire mutations at rates higher than that observed for stromal cells and higher than what would normally be expected for epithelium (Suda et al. 2019)."

Minor comment: The statement from Line 155–156 is very broad; please add discussion/evidence.

Response: We appreciate this comment and have revised this section to provide additional explanation and supporting references Page 6, lines 153-159.

"Identifying recurring molecular alterations in endometrial samples collected over time may help identify women at risk for benign or malignant conditions. Studies have shown that genomic and epigenetic changes in endometrial tissue may lead to the clinical diagnosis. In a study they have shown that aberrant DNA methylation patterns and somatic mutations identified in benign endometrial biopsies correlated with future development of endometrial cancer (Multinu et al. 2020). Another study indicated molecular analysis of endometrial biofluids may improve early detection, risk stratification, and monitoring of women with endometrial hyperplasia who are at risk of developing endometrial cancer (Weng et al. 2022)."

Minor comment: Some additional background summary/introduction of the pathologies discussed in this review might be appropriate.

Response: We have expanded the summary section to provide additional background on the relevant pathologies discussed in this review, including endometriosis and adenomyosis, to improve the overall structure and readability of the manuscript Pages 15-16, lines 406-443.

See Summary above for Reviewer 1: Comment 3.

Reviewer: 2
This review summarizes the recent advancement about the development of endometrial epithelium during physiological and pathological conditions and emphasizes on the mutational landscape. It is an excellent review for the general field.

I have one minor comment. The title of the review is about endometrium. But the paper just starts with the introduction of endometrial epithelium. It has been reported that the epithelial

compartment has more mutations. But for readers in the general field, I think a brief explanation about why focus on epithelium would be more logically sounding.

Response: We thank the reviewer for this kind comments and suggestions. We have revised the title as described earlier and included a brief explanation of why the focus of this review is on the endometrial epithelium in the introduction **Page 3, Lines 55-61**

First revision: MHR-25-0360.R1

Associate Editor

Overall, the authors were receptive to the reviewers' comments and edited the manuscript accordingly. Regarding the authors response to my comment about their focus on epithelial cell mutations, I would like to refer them to a new BioRxiv preprint, which indicates that while the majority of somatic mutations are located in epithelial cells, there are few that are detected in the stromal compartment. Though their revised statement is still correct, inclusion of these finding could strengthen their claims.
https://www.biorxiv.org/content/10.64898/2026.02.25.708037v1.full

Editor-in Chief

The manuscript has been revised to the full satisfaction of the Editorial Team and is accepted for publication. There is one suggested final refinement that can be be dealt with during production (we refer the Authors to the comment of the Associate Editor). We congratulate the Authors and all those involved.

[Editorial Office note: The Associate Editor's comment was addressed by the authors at a final internal checking stage]
